# Histamine H4 Receptor Agonism Induces Antitumor Effects in Human T-Cell Lymphoma

**DOI:** 10.3390/ijms23031378

**Published:** 2022-01-26

**Authors:** Mariángeles Clauzure, Mónica A. Táquez Delgado, Jude M. Phillip, Maria V. Revuelta, Leandro Cerchietti, Vanina A. Medina

**Affiliations:** 1 Laboratory of Tumor Biology and Inflammation, Institute for Biomedical Research (BIOMED), School of Medical Sciences, Pontifical Catholic University of Argentina (UCA), National Scientific and Technical Research Council (CONICET), Buenos Aires 1107, Argentina; mclauzure@gmail.com (M.C.); monicatqzdlgd@gmail.com (M.A.T.D.); 2 Faculty of Veterinary Science, National University of La Pampa (UNLPam), General Pico 6360, Argentina; 3 Hematology and Oncology Division, Department of Medicine, Weill Cornell Medicine, New York, NY 10065, USA; jphillip@jhu.edu (J.M.P.); mvr2002@med.cornell.edu (M.V.R.); lec2010@med.cornell.edu (L.C.)

**Keywords:** T-cell lymphoma, panobinostat, histamine, H4R isoforms, proliferation, apoptosis

## Abstract

The discovery of the human histamine H4 receptor (H4R) has contributed to our understanding of the role of histamine in numerous physiological and pathological conditions, including tumor development and progression. The lymph nodes of patients with malignant lymphomas have shown to contain high levels of histamine, however, less is known regarding the expression and function of the H4R in T-cell lymphoma (TCL). In this work we demonstrate the expression of H4R isoforms (mRNA and protein) in three human aggressive TCL (OCI-Ly12, Karpas 299, and HuT78). Histamine and specific H4R agonists (VUF8430 and JNJ28610244) significantly reduced cell viability in a dose-dependent manner (*p* < 0.05). The combined treatment with the H4R antagonist (JNJ7777120, 10 µM) reversed the effects of the H4R ligands. Importantly, we screened a drug repurposing library of 433 FDA-approved compounds (1 μM) in combination with histamine (10 μM) in Hut78 cells. Histamine produced a favorable antitumor effect with 18 of these compounds, including the histone deacetylase inhibitor panobinostat. Apoptosis, proliferation, and oxidative stress studies confirmed the antitumoral effects of the combination. We conclude that the H4R is expressed in TCL, and it is involved in histamine-mediated responses.

## 1. Introduction

T-cell lymphomas (TCL) constitute a heterogeneous group of non-Hodgkin lymphomas (NHL) with a complex diagnosis and relatively low incidence, representing around 10–15% of NHL in the Western world. Lymphomas that arise from mature T cells can be categorized together under the general term peripheral TCL (PTCL). Based on the World Health Organization (WHO), they are divided into three categories (nodal, extranodal, and leukemic) and are classified in subtypes that include PTCL-NOS (not otherwise specified), anaplastic large cell lymphoma (ALCL), angioimmunoblastic T-cell lymphoma (AITL), and natural killer/T-cell lymphoma (NKTCL) among other rare diseases. Cutaneous TCL (CTCL) are a subclass of extranodal lymphomas that arise within the skin, including Mycosis fungoides and Sézary syndrome [1,2,3].

Although there are various therapeutics currently available, durable disease control is challenging, especially in advanced-stage TCL that is associated with a poor prognosis. Histone deacetylase inhibitors (HDACi) are an emerging class of antitumor agents with the ability to regulate transcriptional and gene expression patterns and cytotoxicity [4,5,6]. HDACi have been intensively investigated as potential drug targets in TCL and other hematologic malignancies [5,6,7,8]. A better knowledge of the tumor biology will not only enable a better classification of the disease but also may provide new therapeutic targets and strategies for single or combination therapies.

Histamine is a biogenic amine involved in numerous pathophysiological conditions, including the regulation of hematopoiesis and hematological malignancies [9,10,11]. The histamine H4 receptor (H4R) is the last discovered member of the family of histamine receptors and it is present in hematopoietic and immune cells. The H4R is highly expressed in the bone marrow, spleen, and in other tissues of the gastrointestinal tract, testes, kidney, lung [12,13,14,15]. Alternative splicing of the human *H4R* gene generates possible splice variants. The complete sequence encodes 390 amino acids of full-length H4R_(390)_ protein. Shorter isoforms do not bind histamine and might elicit modulatory effects on H4R signaling. However, their pathophysiological relevance is still unknown [14,16,17]. Interestingly, H4R functional expression was further described in different types of tumors, and preclinical studies show that its agonistic activation regulates numerous antitumor-associated effects [10,11,12,18,19,20,21]. In addition, histamine is able to selectively modulate the effects of anticancer therapies, including ionizing radiation, chemotherapy—i.e., doxorubicin, and immunotherapy [20,21,22,23,24,25]. In line with this, histamine dihydrochloride administration in combination with IL-2 has been approved in Europe for the treatment of adults with acute myeloid leukemia (AML) [25,26].

Histamine is the endogenous receptor agonist and binds to the H4R with high affinity. However, this biogenic amine has cross-reactivity with all four histamine receptor subtypes. Soon after H4R discovery, numerous H4R ligands (agonists and antagonists) were developed attempting to identify the pharmacological profile and function of the H4R. The indole carboxamide compound JNJ7777120 was the first reported selective and potent H4R antagonist, and it has been broadly used as the reference pharmacological agent to determine the role of H4R in several in vitro and in vivo experimental models of disease [10,12,14,15]. Among the specific agonists, VUF8430 and 4-methylhistamine are the most widely used selective full agonists at the H4R. The experimental JNJ28610244 compound has demonstrated excellent potency and selectivity for the H4R, and numerous studies support its use as a H4R agonist [12,14,15]. Until now, only H4R antagonists, which include toreforant (JNJ38518168) and adriforant (ZPL-3893787) are being evaluated in clinical settings for their potential therapeutic applications in immune-related diseases [12].

Although it was previously reported that histamine levels in lymph nodes of patients with malignant lymphomas (Hodgkin’s disease and NHL) are higher compared to control individuals [27], limited data exist on the role of histamine and H4R in TCL.

The aim of the present work was to investigate the expression of the H4R in TCL, and to evaluate the potential antitumor effects of histamine and H4R ligands. We show that H4R isoforms are expressed in TCL and are involved in histamine-mediated responses. Histamine could be an attractive compound for its use as a single agent or in combination with HDACi for the treatment of TCL.

## 2. Results

### 2.1. H4R Expression in TCL

**Figure 1:**
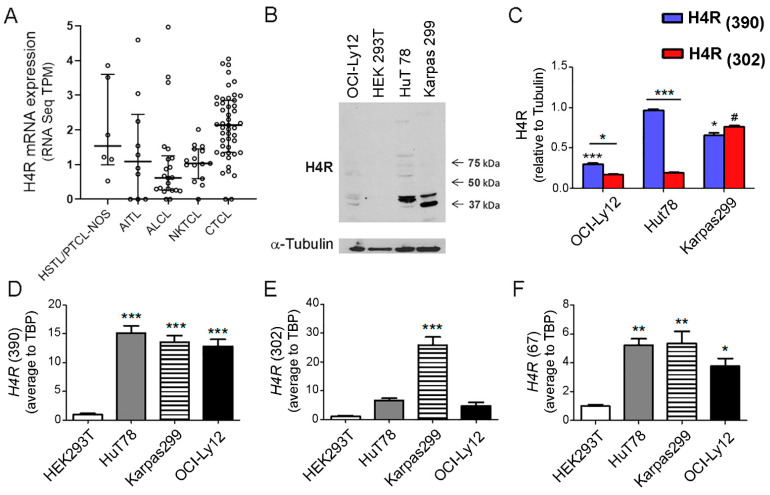
H4R expression in TCL. (**A**) *H4R* mRNA expression (ENST00000256906.5 transcript variant 1, full length) in tumors of TCL patients. The transcriptional datasets of tumors from patients with AITL (accession number: SRP029591, *n* = 10) [28], ALCL (accession number: SRP044708, *n* = 22) [29], Hepatosplenic TCL (HSTL)/PTCL-NOS (accession number: SRP039591, *n* = 6) [30], NKTCL (accession number: SRP049695, *n* = 15) [31], and CTCL (accession number: SRP139926, *n* = 47) [32] were obtained from the NCBI Sequence Read Archive and analyzed as indicated in Materials and Methods. TPM: transcripts per million. For dot plots the center line is the median with the interquartile range. (**B**) OCI-Ly12, HEK293T, HuT78, and Karpas299 cells were pre-incubated for 24 h in serum-free medium. HEK293T cells were used as a negative control of H4R expression. Representative Western blot of H4R and α-Tubulin of whole cellular lysates from OCI-Ly12, HEK293T, HuT78, and Karpas299 cells. (**C**) The expression of molecular species compatible with the H4R_(390)_ and H4R_(302)_ isoforms relative to α-Tubulin were quantified using ImageJ software and plotted as arbitrary units (AU). Data are the mean ± SEM of five independent samples. * indicates *p* < 0.05 and *** indicates *p* < 0.001 compared with *H4R*_(390)_ in HuT78 cells. # indicates *p* < 0.05 compared with *H4R*_(302)_ in HuT78 cells. (**D**) Quantitative real-time RT-PCR of *H4R*_(390)_ isoform mRNA levels. (**E**) Quantitative real-time RT-PCR of *H4R*_(302)_ isoform mRNA levels. (**F**) Quantitative real-time RT-PCR of *H4R*_(67)_ isoform mRNA levels. Measurements were performed in triplicates for each condition and cell line and data are expressed as mean ± SEM (*n* = 3 independent experiments). * indicates *p* < 0.05, ** indicates *p* < 0.01, *** indicates *p* < 0.001 compared with HEK293T.

To date, the evidence of H4R expression in TCL has remained unknown. Using RNA-seq available data from different studies we surveyed the mRNA levels of the *H4R* gene in tumors of TCL patients [28,29,30,31,32]. As shown in Figure 1A, a relatively low expression of the full length isoform *H4R*_(390)_ was found in CTCL and the most common subtypes of PTCL (Figure 1A). The splice variant isoform *H4R*_(302)_ was additionally detected only in a few samples (Figure S1).

The expression of H4R protein was confirmed by Western blot in HuT78, Karpas299, and OCI-Ly12 TCL cells. The anti-H4R antibody (ab97487) recognizes regions within amino acids 1–52, which is present in the H4R protein isoforms. As shown in Figure 1B, Western blot demonstrated the presence of a diverse molecular weight species of the H4R that are compatible with the full length isoform H4R_(390)_ (44 kDa) and the splice variant isoform H4R_(302)_ (34 kDa) [15,16,17], and the pattern of their expression varied according to the cell lines. The specificity of the H4R antibody was evaluated by Western blot, using HEK293 cell line, which is devoid of H4R and thus, it was used as a negative control of H4R expression [16,17,33]. HuT78 cells exhibited the highest level of H4R_(390)_ isoform compared to Karpas299 and OCI-Ly12 TCL cells while Karpas299 has significantly higher levels of H4R_(302)_ than the other TCL cells. Both HuT78 and OCI-Ly12 cells show significantly increased levels of H4R_(390)_ compared to H4R_(302)_ isoform (Figure 1B,C).

We further investigated the expression of *H4R* isoforms at the mRNA level. The primers were designed to amplify individually each *H4R* isoform’s transcript. High expression of the *H4R*_(390)_ isoform mRNA at similar levels was observed in HuT78, Karpas299, and OCI-Ly12 TCL cells (Figure 1D). In contrast, the *H4R*_(302)_ isoform mRNA expression exhibited different levels, depending on the cell line (Figure 1E). Karpas299 cells significantly showed the highest expression of *H4R*_(302)_ isoform compared to HuT78 and OCI-Ly12 cells (Figure 1E). Truncated *H4R*_(67)_ isoform mRNA showed the lowest expression level in all cell lines compared to the other two isoforms (Figure 1F).

### 2.2. Effect of Histamine and H4R Ligands on the Viability of TCL Cell Lines

**Figure 2:**
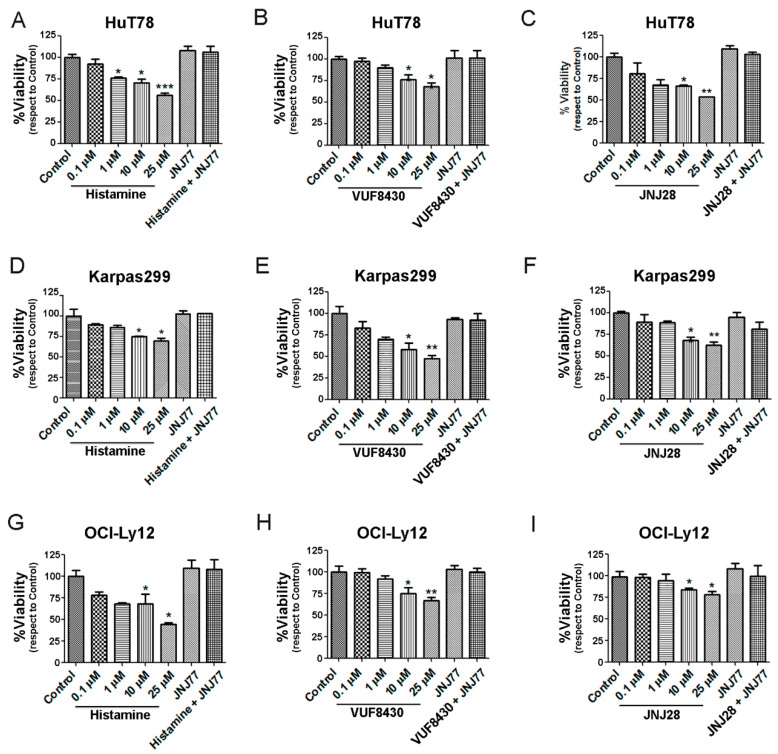
Effect of histamine and H4R agonists on the viability of HuT78, Karpas299, and OCI-Ly12 cells. Cells were pre-incubated for 24 h in serum-free RPMI medium and then treated as indicated: HuT78 (**A**–**C**), Karpas299 (**D**–**F**), OCI-Ly12 (**G**–**I**) cells were left untreated (control) or were treated with histamine, JNJ28, VUF8430 (0.1, 1, 10 and 25 µM) or JNJ77 (10 μM), and histamine (10 µM), JNJ28 (10 µM), VUF8430 (10 µM) combined with JNJ77 (10 μM) for 48 h in complete medium and viability was evaluated by Cell Titer Blue Assay. Measurements were performed in triplicates for each condition and cell line and data are expressed as mean ± SEM (*n* = 3 independent experiments). * indicates *p* < 0.05, ** indicates *p* < 0.01, *** indicates *p* < 0.001 compared with Control.

We next investigated whether histamine and the specific H4R agonists JNJ28610244 (JNJ28) and VUF8430 can modulate cell viability. Results demonstrate that histamine, JNJ28, and VUF8430 significantly decreased cell viability in a dose-dependent manner in HuT78 cells after 48 h of treatment. Similar results were found in H4R agonist-treated Karpas299 (Figure 2D–F) and OCI-Ly12 cells (Figure 2G–I). In all cell lines, treatment with the H4R antagonist JNJ7777120 (JNJ77), added 30 min before any other treatment, completely reversed the effect of the H4R agonists on cell viability.

### 2.3. Effect of Histamine and H4R Ligands on the Apoptosis of TCL Cell Lines

**Figure 3:**
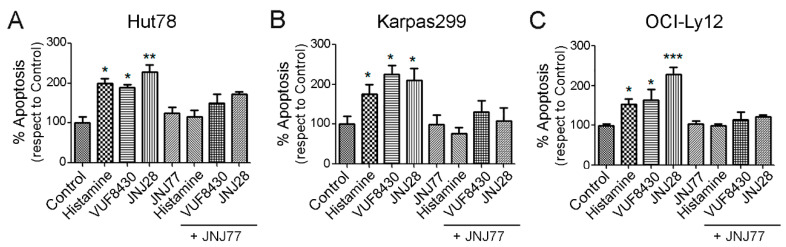
Modulation of apoptosis in HuT78, Karpas299, and OCI-Ly12 cells by histamine and H4R agonists. (**A**) HuT78, (**B**) Karpas299, (**C**) OCI-Ly12 cells were pre-incubated for 24 h in serum-free RPMI medium and then were left untreated (control) or were treated with histamine, JNJ28 or VUF8430 (10 µM) and/or JNJ77 (10 μM) for 48 h in complete medium, and apoptosis was evaluated by Caspase-Glo 3/7 Assay. Measurements were performed in triplicates for each condition and cell line and data are expressed as mean ± SEM (*n* = 3 independent experiments). * indicates *p* < 0.05, ** indicates *p* < 0.01, *** indicates *p* < 0.001 compared with Control.

To corroborate the biological significance of H4R in TCL cell lines, we next analyzed the apoptotic regulation induced by histamine, JNJ28, and VUF8430. The treatment of HuT78 cells with histamine (10 µM), JNJ28 (10 µM), or VUF8430 (10 µM) for 48 h increased the percentage of apoptotic cells evaluated by Caspase-Glo 3/7 Assay (Figure 3A). The apoptotic effect was reversed when JNJ77 (10 µM) was added to the medium 30 min before H4R agonists’ treatment. Similar results were observed in Karpas299 (Figure 3B) and OCI-Ly12 cells (Figure 3C).

### 2.4. Modulation of H4R mRNA Expression in HuT78 Cells by Histamine and Specific H4R Agonists

According to the Western blot analysis, HuT78 cells exhibited the highest expression level of the full length and biologically active isoform of the H4R (390 aa) compared to the other cell lines. Therefore, we decided to deepen the evaluation of the therapeutic efficacy of histamine and H4R’s ligands in these TCL cells.

**Figure 4:**
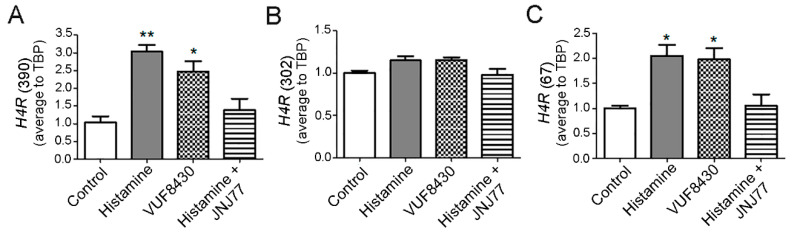
Regulation of *H4R* isoforms’ mRNA expression in HuT78 cells by histamine and H4R agonists. HuT78 cells were pre-incubated for 24 h in serum-free RPMI medium and then were left untreated (control) or were treated with histamine (10 μM), VUF8430 (10 μM), and histamine (10 μM) plus JNJ77 (10 μM) for 30 min. (**A**) Quantitative real-time RT-PCR of *H4R*_(390)_ isoform mRNA levels. (**B**) Quantitative real-time RT-PCR of *H4R*_(302)_ isoform mRNA levels. (**C**) Quantitative real-time RT-PCR of *H4R*_(67)_ isoform mRNA levels. Measurements were performed in triplicates for each condition and cell line and data are expressed as mean ± SEM (*n* = 3 independent experiments). * indicates *p* < 0.05, ** indicates *p* < 0.01 compared with Control.

To elucidate whether H4R ligands’ treatment could modulate *H4R* expression, the mRNA expression of the three isoforms were analyzed upon histamine (10 µM) and VUF8430 (10 µM) treatment for 30 min in HuT78 cells. As depicted in Figure 4A, histamine and VUF8430 increased *H4R*_(390)_ isoform’s expression, an effect that was blocked with the treatment with JNJ77 (10 µM). A similar pattern of expression was observed for the *H4R*_(67)_ isoform (Figure 4C). However, no significant changes were observed in the expression of *H4R*_(302)_ isoform (Figure 4B).

### 2.5. Therapeutic Benefit for the Combination Therapy with Histamine and Histone Deacetylase Inhibitors

**Figure 5:**
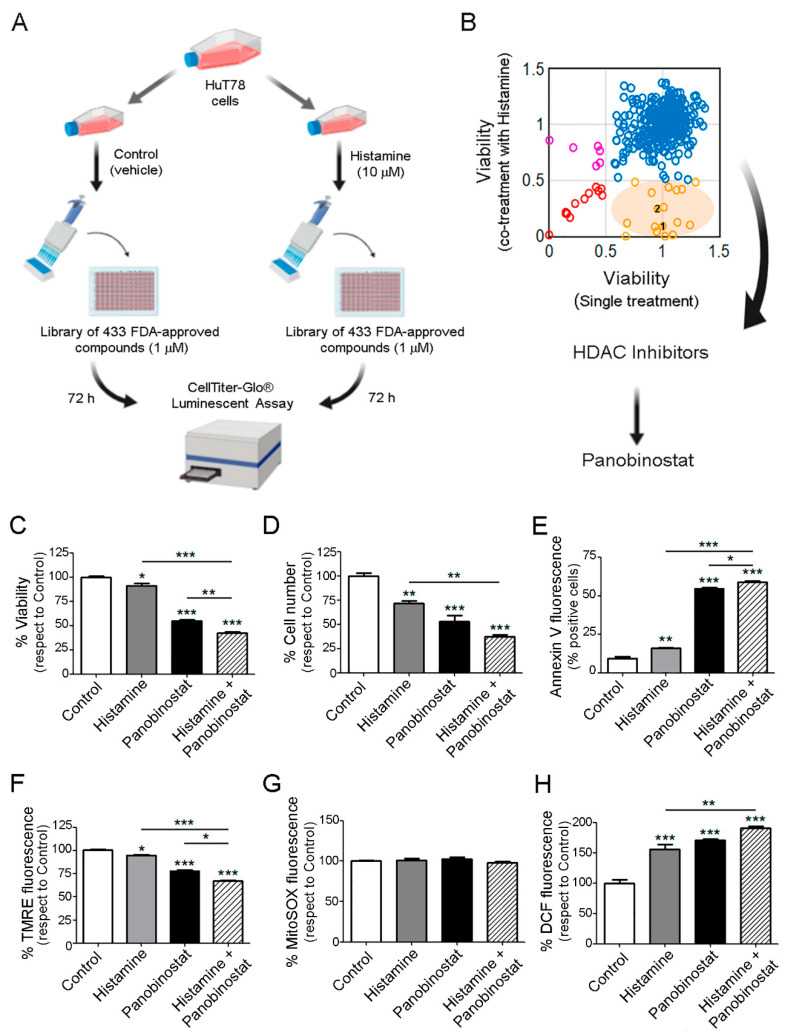
Screening of a library of 433 FDA-approved compounds. Effect of the combination of histamine and panobinostat on HuT78 cell proliferation, apoptosis, and ROS production. HuT78 cells were pre-incubated for 24 h in serum-free RPMI medium and then were treated with the FDA-approved drug (single treatment) or were treated with the combination of histamine (10 μM) plus FDA-approved compound for 72 h in triplicates for each condition. (**A**) Experimental design. (**B**) Results of the screening experiment, showing potential favorable effects with the combined treatment of histamine plus HDAC inhibitors: (1) panobinostat, (2) belinostat. (**C**) HuT78 cells were pre-incubated for 24 h in serum-free RPMI medium and then were left untreated (control) or were treated with histamine (10 µM) and/or panobinostat (1 µM) for 48 h in complete medium, as indicated. Cell viability was evaluated by Cell Titer Blue Assay. (**D**) The cell number was counted using a Neubauer chamber. (**E**) Percentage of Annexin-V positive cells. (**F**) Mitochondrial membrane potential measured by the TMRE fluorescence determination. (**G**) MitoSOX fluorescence (5 µM) was measured using a microplate reader. (**H**) DCF fluorescence (10 µM DCFH-DA) was measured using a microplate reader. Measurements were performed in quintuplicate (**C**) and triplicates (**D**–**H**) for each condition and cell line and data are expressed as mean ± SEM (*n* = 3 independent experiments). * indicates *p* < 0.05, ** indicates *p* < 0.01, *** indicates *p* < 0.001 compared with Control.

We next exposed HuT78 cells to a drug screening library consisting of 433 compounds (SelleckChem) (Supplementary Table S1). Cells were treated with single agents (at 1 μM concentration) or the compounds in combination with histamine (10 μM) for 72 h (Figure 5A). We observed a favorable antitumor effect upon co-treatment with 18 of these compounds showing differential activity in the presence of histamine, including the HDACi, panobinostat (1) and belinostat (2) (Figure 5B). The HDACi panobinostat showed a higher response when combined with histamine compared to belinostat and thus, we continued exploring the efficacy of this combination in further experiments.

The antitumoral effect of the combination was confirmed evaluating apoptosis, proliferation, and oxidative stress parameters. Results demonstrated that histamine (10 µM) and panobinostat (1 µM) significantly decreased HuT78 cell viability and count after 48 h of treatment. Moreover, histamine enhanced panobinostat-induced effect on cell viability and proliferation (Figure 5C,D). Similar results were found with the combination of histamine and vorinostat (1 µM), another HDACi (Figure S2). In addition, the treatment of HuT78 cells with histamine further increased the percentage of apoptotic cells induced by panobinostat, evaluated by flow cytometric analysis of Annexin-V staining (Figure 5E). Accordingly, treatment with histamine, panobinostat, and the combination of both drugs to a higher extent, reduced the mitochondrial transmembrane potential evaluated by TMRE staining (Figure 5F).

Finally, we evaluated whether histamine in combination with HDACi could regulate the ROS levels in HuT78. As it is shown in Figure 5G, the mitochondrial ROS levels measured by MitoSOX staining did not change upon histamine and/or panobinostat treatment. Different results were found when the cellular ROS levels were measured by using the fluorescent probe DCFH-DA. Treatment of HuT78 cells with histamine (10 µM) or panobinostat (1 µM) for 48 h increased the percentage of cellular ROS levels. Interestingly, the combination of histamine and panobinostat showed a potentiating effect increasing even more the cellular ROS levels (Figure 5H).

## 3. Discussion

At the beginning of the millennium, the H4R was discovered independently by numerous scientific groups of the academy and industry. H4R is predominantly expressed in cells of the immune system such as mast cells, basophils, eosinophils, dendritic cells, Natural Killer cells, and T lymphocytes, and it exhibits functional implications in inflammatory diseases and immunomodulatory pathways [12,13,14,17,34,35,36,37,38,39,40]. The human *H4R* gene is mapped on chromosome 18q11.2, and it is made up of three exons separated by two large introns. *H4R* encodes a 390 amino acid protein with an estimated molecular weight of 44 kDa. Alternative splicing of the gene can occur, and two lower molecular weight isoforms have been described. The *H4R*_(302)_ isoform of 302 amino acids, has an interior deletion between transmembrane domains II and IV and a molecular weight of 31–34 kDa. The truncated *H4R*_(67)_ isoform of 67 amino acids consists of the transmembrane domains I and part of the II [15,17,36].

In the present work, we show the mRNA expression of *H4R* in human TCL samples. We validated the H4R expression in HuT78, Karpas299, and OCI-Ly-12 TCL cell lines. The full length H4R and the two alternatively spliced isoforms were differentially expressed in the three TCL cells. Western blot analysis shows the predominant expression of H4R_(390)_ and H4R_(302)_ isoforms. Additional bands were occasionally detected, which could likely represent oligomerization forms, unglycosylated or heavily glycosylated forms of the H4R [15]. In agreement with these results, qPCR showed that the mRNA encoding *H4R*_(390)_ full length isoform and the *H4R* splice variants are differentially expressed in the TCL cell lines. The *H4R*_(390)_ was highly expressed in the three cell lines, while splice variant mRNA *H4R*_(302)_ was more abundant in the Karpas299 cells compared to the other cells, which is in line with the protein expression levels determined by Western blot. The lower abundance of the mRNA of the truncated splice variant *H4R*_(67)_ in all cell lines could be related to the abundant expression of full length form [17]. The specificity of both assays was checked using HEK293 cells, which do not endogenously express H4R [16,17]. Very few reports studying the differential expression of H4R isoforms are available, especially in native, non-genetically modified human cells. A dominant negative effect of the splice variants was described by other authors [15,17]. Therefore, it is possible that in different cells or under diverse pathophysiological conditions, the H4R splice variants could be expressed more or less abundantly, modulating the expression and function of the full-length isoform. Future studies should be performed to address these important questions.

A more detailed mRNA expression analysis demonstrated that *H4R*_(390)_ and *H4R*_(67)_ were upregulated while *H4R*_(302)_ isoform exhibited no changes upon histamine and H4R agonist treatments in HuT78, which might offer insight into the functional role and regulation of this receptor.

To explore the role of histamine-induced activation of the H4R in TCL cells, we investigated its effects on cell viability and apoptosis using a pharmacological approach. Histamine and specific H4R agonists produced a dose dependent decrease in cell viability in the three TCL cell lines. This antitumor effect is associated with an increase in apoptotic cell death. Both the proapoptotic and the cytotoxic effects were prevented by the combined treatment with the H4R antagonist JNJ7777120, confirming that histamine-induced actions are primarily mediated by the H4R. Recent evidence shows that histamine through H4R plays important roles at a variety of stages during tumor development and progression, producing protumoral or antitumoral effects depending on the cancer cell type. Although in colon cancer the activation of H4R induced cell proliferation and VEGF expression, in other cancer subtypes including, melanoma, cholangiocarcinoma, oral squamous cell carcinoma, and breast, esophageal and lung cancers, histamine via H4R reduced proliferation, epithelial to mesenchymal transition, or tumor spread [11,18,38,39,40,41,42]. The later effects are in agreement with the findings observed in TCL.

Martnet et al., (2015) reinforced the hypothesis that histamine is involved in lymphoma progression. In vivo histamine treatment reduced the tumor growth of murine lymphoma developed with EL-4 TCL cells, inducing the intratumoral accumulation of maturated dendritic cells [43]. Furthermore, targeting NOX2 by histamine treatment produced less immunosuppressive intratumoral myeloid derived suppressor cells (MDSCs) and reduced the growth of EL-4 lymphoma while improved the antitumor efficacy of immune checkpoint blockade with antibodies against the programmed cell death receptor 1 (PD-1) and the PD-1 ligand (PD-L1) in EL-4-bearing mice [44]. In vitro treatment of EL-4 cells with histamine showed a significant increase in the cell growth at micromolar concentration [45] while another study demonstrated no alterations in cell proliferation upon histamine treatment in vitro, suggesting that in this murine model of lymphoma the anti-tumor properties of histamine may comprise the targeting of MDSCs [44].

The prognosis of advanced TCL is poor and characterized by aggressive behavior with little response to chemotherapy [1,2,3]. Therefore, to improve the therapeutic benefit for these cancer patients, it is necessary not only to develop new effective treatments, but also to optimize available therapies. The selection of an appropriate therapy requires a multidisciplinary approach. Numerous preclinical and clinical studies show that histamine enhances the efficacy of antitumoral therapies such as ionizing radiation, chemotherapy, and immunotherapy in different cancer types, supporting the rationale for the use of combination therapy with histamine in clinical settings [11,21,43,45,46,47,48,49,50,51].

Several clinical trials were performed with IL-2 immunotherapy combined with histamine for solid neoplastic diseases and hematopoietic cancers with promising results. This combination therapy improved leukemia-free survival and was approved in Europe for the treatment of adults with AML [25,26,46,47,48,49,50]. A study in Chinese healthy volunteers demonstrated the safety profile and pharmacokinetic properties of a single dose of histamine (0.5 or 1 mg) [52,53].

A strategy that could help to identify new medical indications for approved drugs consists of drug repurposing [54]. To identify repurposed drug candidates, in this work we screened 433 FDA-approved compounds for the antitumor efficacy against the HuT78 cell line. Results indicate that histamine produced a favorable effect with 18 compounds, including two members of the family of HDACi, panobinostat and belinostat [55]. We continued the analysis with the former, considering that it produced the highest antitumoral effect when combined with histamine. Based on the promising preclinical findings in both hematologic malignancies and solid tumors, panobinostat and other HDACi have undergone a rapid development of numerous clinical trials, either as individual agents or in combination with other therapies [4,5,6,55]. Currently, the FDA has only approved HDACi as a treatment for hematologic malignancies [56,57,58,59,60]. The drugs vorinostat and belinostat have been approved by the FDA for the treatment of TCL. Panobinostat is approved in several countries for its use in combination with bortezomib and dexamethasone in patients with multiple myeloma [7,8].

HDACi produces multiple antitumor effects that include cell cycle arrest, induction of apoptosis, inhibition of angiogenesis, and decreased invasion and metastasis [8,61]. The complementary studies were in agreement with the results obtained in the metabolic assay performed in the screening. The combination of histamine with panobinostat in HuT78 TCL cells enhanced the antitumor effect of panobinostat, increasing cell cytotoxicity and apoptosis, and decreasing the membrane potential. These results are probably related to the modulation of oxidative stress. Similar findings were obtained with the combination of histamine and vorinostat. Further studies are needed to fully understand the mechanisms involved in histamine-mediated effects.

## 4. Materials and Methods

### 4.1. Chemicals

Histamine (Sigma Chemical Co., St. Louis, MO, USA); H4R agonist: VUF8430 (Tocris Bioscience, Ellisville, MO, USA); JNJ28610244 (JNJ28) (Janssen Research & Development, San Diego, CA, USA). H4R antagonist: JNJ7777120 (JNJ77) (Janssen Research & Development). Panobinostat were from Selleckchem (Houston, TX, USA).

### 4.2. Transcriptional Datasets Analyses

RNA-seq data from tumors of PTCL patients were obtained from the NCBI Sequence Read Archive (SRA), accession numbers: SRP029591 [28], SRP044708 [29], SRP039591 [30], SRP049695 [31], SRP139926 [32].

Data was filtered and quality checked with fastp v0.20.0 [62]. Transcripts ENST00000256906.5 (variant 1, full length) and ENST00000426880.2 (variant 2, short isoform) were quantified with Salmon v1.5.2 [63], and imported into R v4.1.0 with tximport v1.20.0 [64] for scaled TPM visualization. Results are expressed as log (TPM+1).

### 4.3. Cultured Cells

HuT78 cells (human CTCL cell line, Sézary Syndrome) and HEK293T (human cell line originally derived from human embryonic kidney cells) were obtained from ATCC. OCI-Ly12 (human PTCL-NOS, cell line) was obtained from the Ontario Cancer Institute. Karpas299 (human TCL cell line, ALCL) was obtained from the DMSZ. HEK293T cells were cultured in DMEM (Life Technologies, GIBCO BRL, Rockville, MD, USA) supplemented with 10% FCS, 100 U/mL penicillin and 100 µg/mL streptomycin (Life Technologies, GIBCO BRL, Rockville, MD, USA). Other cell lines were cultured in RPMI-1640 medium, supplemented with 10% FCS and 2 mM glutamine (complete medium). Cultures were grown at 37 °C in a humidified air atmosphere containing 5% CO_2_.

### 4.4. Reverse Transcription and Quantitative Real-Time PCR (qRT-PCR) for H4R Isoforms

Cells were cultured as indicated above. After incubation, RNA was isolated from cell lines using TRIzol reagent (Invitrogen) and measured using the Nanodrop 1000 spectrophotometer. Equal amounts of RNA were converted to cDNA using Verso cDNA Synthesis Kit (Thermo scientific, Waltham, MA, USA). The synthesized cDNAs were used immediately for PCR amplification or stored at −80 °C for later use. qRT-PCR was performed in 384-well plates using a 7900 HT fast real-time PCR system (Applied Biosystems, Waltham, MA, USA). *TBP* (Tata Box Binding Protein) was used as an internal control. Primer sequences for PCR were as follows: *H4R*_(390)_*,* 5′-ACTTGGCCATCTCTGACTTCT-3′ (forward) and 5′-CATTCGAACAGCGTGTGAGG-3′ (reverse); *H4R*_(302)_ 5′-TGACTTCTTTGTGGTTTCAGAGT-3′ (forward) and 5′-GGCAAGGATGTACCATTCCG-3′ (reverse); *H4R*_(67)_ 5′-CTCTGACTTCTTTGTGGGTGTCT-3′ (forward) and 5′-AACGGCCACCATCAGAGTAAC-3′ (reverse). qRT-PCR reactions were carried out in triplicates (intra- and inter-assays by triplicate). Fold change in gene expression was calculated using the ΔΔCT method.

### 4.5. Protein Extraction

Cells were incubated as above indicated, washed twice with cold PBS, scraped with cold extraction buffer (10 mM Tris pH 7.4, 100 mM NaCl, 0.1% SDS, 0.5% sodium deoxycholate, 1% Triton X-100, 10% glycerol) containing the protease inhibitor cocktail (5 mL of cocktail/20 g of cell extract) plus phosphatase inhibitors (2 mM Na_3_VO_4_, 1 mM NaF and 10 mM Na_2_PO_7_), and centrifuged at 14,000× *g* for 20 min at 4 °C. The supernatant was stored at −80 °C until use. The protein concentration was measured by using the method of BCA protein assay.

### 4.6. Immunoblotting

Total protein extracts (30–50 µg of proteins) were separated on a denaturing SDS-PAGE (15%) and transferred to nitrocellulose membranes. Membranes were blocked with BSA 5% in TBS 1 h and then incubated ON with primary polyclonal antibody against H4R (ab97487; Abcam, Cambridge, UK) (dilutions 1:1500 in TBS plus Tween-20, 0.05% *v*/*v*). The membranes were washed three times with TBS plus Tween-20 (0.05% *v*/*v*) for 5 min and incubated for 1 h with goat IgG anti-rabbit antibody coupled to horseradish peroxidase (sc-2004, Santa Cruz Biotechnology, Dallas, TX, USA) (dilution 1:5000 in TBS plus Tween-20, 0.05% *v*/*v*), washed three times with TBS plus Tween-20 and developed. As internal controls, membranes were re-incubated for 3 h with primary monoclonal antibody against α-tubulin (T9026; Sigma) (dilution 1:5000 in TBS plus Tween-20, 0.05% *v*/*v*), washed three times as above indicated, and then incubated for 1 h with goat IgG anti-mouse antibody coupled to horseradish peroxidase (sc-2005, Santa Cruz Biotechnology) (dilution 1:5000 in TBS plus Tween-20, 0.05% *v*/*v*). We used ECL Western Blotting Substrate (Pierce Biotechnology) according to the manufacturer’s instructions and the blots were visualized by autoradiography. Quantitative densitometry analysis of Western blot bands was performed employing ImageJ version 10.2 (NIH, Bethesda, MD, USA). The normalized relative densities were calculated relative to the expression of α-tubulin.

### 4.7. Cell Viability Assay

Cell viability was measured with a fluorometric resazurin reduction method (CellTiter-Blue; Promega, Madison, WI, USA). Briefly, 5 × 10^5^ cells/mL were seeded at a final volume of 0.1 mL in 96-well flat-bottom microtiter plates and were treated as indicated in results. Fluorescence (560Ex/590Em) was determined using a luminometer (NovoStar microplate reader, BMG Labtech, Ortenberg, Germany).

### 4.8. Apoptosis Determinations

Cells were seeded into 12-well plates (2.5 × 10^4^ cells/well) and treated with 10 μmol L^−1^ histamine, 1 μmol L^−1^ panobinostat or both for 48 h. Phosphatidylserine exposure on the surface of apoptotic cells was determined by staining with Annexin-V FITC and PI (50 μg/mL) (BD Biosciences, San José, CA, USA) by using flow cytometry, according to the manufacturer’s instructions and previously reported. Samples were run on a BD Accuri C6 flow cytometer (BDB), and data were analyzed by using the BD Accuri C6 software (BDB).

Caspase 3 and 7 activity was determined using caspase-Glo 3/7 Assay (Promega, USA) following manufacturer’s instructions. HuT78, OCI-Ly12, and Karpas299 cell lines were treated as indicated in results. Luminescence was measured using the Synergy4 microplate reader (BioTek, Winooski, VT, USA).

### 4.9. Drug Screening Library

Plates with the library of 433 FDA-approved compounds from Selleckchem’s anticancer drug library (Supplementary Table S1). HuT78 cells were seeded into plates (2 × 10^4^ cells/well), and were treated or not with 10 μM of histamine for 72 h. To determine the cell viability, we used the homogeneous method CellTiter-Glo^®^ Luminescent Cell Viability Assay (Promega, USA) following manufacturer’s instructions. It is based on quantification of the ATP present, which signals the presence of metabolically active cells. Luminescence was measured using the Synergy4 microplate reader (BioTek).

### 4.10. Measurement of Mitochondrial and Cellular ROS Levels

Mitochondrial and cellular ROS levels were measured by using fluorescent probes in 96 well black plates (Greiner Bio-One, Leipzig, Germany; 655090) as previously described [65,66]. The cells were cultured and treated as above indicated. To measure mitochondrial ROS levels, at the end of incubation, the medium was changed to Hank’s solution (136.9 mM NaCl, 5.4 mM KCl, 1.3 mM CaCl_2_, 3.7 mM NaH_2_PO_4_, 0.4 mM KH_2_PO_4_, 4.2 mM NaHCO_3_, 0.7 mM MgSO_4_, 5.5 mM D-glucose and 10 mM HEPES) containing 5 µM of MitoSOX (stock prepared as 5 mM solution in DMSO) and incubated at 37 °C in the 5% CO_2_/air incubator for 10 min. Cellular ROS levels were measured by using the fluorescent probe DCFH-DA in Hank’s solution containing 10 µM of the fluorescent probe (stock prepared as 20 mM solution in DMSO) and incubated at 37 °C in the 5% CO_2_/air incubator for 40 min. Then, cells were washed with 0.2 mL of Hank’s solution three times and the fluorescence was measured in a fluorescence plate reader (NOVOstar, BMG LABTECH GmbH, Ortenberg, Germany) with incubation at 37 °C. Filters were Ex = 510 ± 10 nm, Em = 580 ± 10 nm for MitoSOX, and Ex = 510 ± 10 nm, Em = 540 ± 10 nm for DCFH-DA, and readings were performed from the bottom of the plate). As positive control for the technique we used rotenone, a specific inhibitor of mCx-I, for mitochondrial ROS levels, and H_2_O_2_ for cellular ROS levels.

### 4.11. Mitochondrial Transmembrane Potential Determination

The cell-permeant, cationic, red-orange fluorescent dye tetramethylrhodamine ethyl ester (TMRE) (Molecular Probes, Life Technologies Corporation, Carlsbad, CA, USA), which is rapidly sequestered by active mitochondria, was used to evaluate the mitochondrial transmembrane potential. Since dead cells become completely depolarized, we analyzed live-gated cells to detect the decrease in mitochondrial transmembrane potential, which is associated with apoptosis. Cells were incubated at 37 °C for 30 min in the presence of 40 nM TMRE. They were then harvested after washing with PBS, and analyzed by flow cytometry (BD Accuri C6, BDB). The mean fluorescence of untreated cells was set at 100%. CCCP (carbonyl cyanide m-chlorophenyl hydrazone), a mitochondrial oxidative phosphorylation uncoupler, was used as a positive control at a concentration of 20 μM during 30 min.

### 4.12. Statistics

One-way ANOVA and Tukey’s test were applied to calculate significant differences among samples (α = 0.05). All statistical analyses were performed with GraphPad Prism version 7.00 (San Diego, CA, USA).

## 5. Conclusions

Our study provides the first evidence of the expression of the H4R isoforms in human TCL, and demonstrates that this histamine receptor subtype is involved in histamine-mediated antitumor responses. In the different cell lines employed, the H4R ligands produced similar responses in terms of cell viability and apoptosis, which seemed to be independent of the profile of H4R isoforms expressed in each cell line. We hypothesize that the level of expression of the full length functional H4R isoform is enough to trigger the studied antitumoral responses. However, the precise role of the H4R isoforms in cell proliferation needs to be thoroughly explored.

Histamine produces antitumor effects and improves the efficacy of panobinostat thus, this biogenic amine might represent an attractive compound to be used as a single therapy or in combination with HDACi for the treatment of these hematological malignancies.

## Data Availability

The data that support the findings of this study are available from the corresponding author upon reasonable request.

## Supplementary Materials

The following supporting information can be downloaded at: https://www.mdpi.com/article/10.3390/ijms23031378/s1.
