# Supplementary material for: Histamine H4 Receptor Agonism Induces Antitumor Effects in Human T-Cell Lymphoma
Source: Int J Mol Sci. 2022 Jan 26;23(3):1378. doi: 10.3390/ijms23031378 (PMC8836034; doi:10.3390/ijms23031378)
Supplement: Supplementary file 1 [file ijms-23-01378-s001.zip › ijms-1488512-supplementary.pdf]

Supplementary Materials

Table S1: List of a library of 433 FDA-approved compounds.

Number	Drug
1	FG-4592
2	Rizatriptan Benzoate
3	Fulvestrant
4	Tolfenamic Acid
5	Ramelteon
6	Trospium chloride
7	Granisetron HCl
8	A-769662
9	Losartan Potassium (DuP 753)
10	Tolazoline HCl
11	Tenofovir Disoproxil Fumarate
12	Ataluren (PTC124)
13	Candesartan
14	Belinostat (PXD101)
15	Semagacestat (LY450139)
16	NVP-ADW742
17	NSC 319726
18	Icotinib
19	Ilomastat (GM6001, Galardin)
20	Rivaroxaban
21	STF-118804
22	Rimonabant
23	FLI-06
24	Sitaxentan sodium
25	ABT-263 (Navitoclax)
26	Brinzolamide
27	Nilotinib (AMN-107)
28	Tranylcypromine (2-PCPA) HCl
29	Tandutinib (MLN518)
30	Zebularine
31	MLN8054
32	PR-619
33	Roxatidine Acetate HCl

34	GSK1904529A
35	Atorvastatin Calcium
36	SNS-032 (BMS-387032)
37	Naltrexone HCl
38	Ganetespib (STA-9090)
39	CGS 21680 HCl
40	Vemurafenib (PLX4032, RG7204)
41	Pancuronium dibromide
42	Loratadine
43	PNU-120596
44	Ruxolitinib (INCB018424)
45	GW3965 HCl
46	AZD6482
47	SB705498
48	Safinamide Mesylate
49	Tenofovir
50	P22077
51	Aloxistatin
52	NSC697923
53	Apixaban
54	ML347
55	Irinotecan HCl Trihydrate
56	SSR128129E
57	VX-765
58	Ferrostatin-1 (Fer-1)
59	Rotundine
60	MM-102
61	PF-3845
62	OTX015
63	Ibrutinib (PCI-32765)
64	4E1RCat
65	Tofacitinib (CP-690550,Tasocitinib)
66	PHA-793887
67	WZ811
68	Allopurinol
69	HC-030031
70	Telmisartan
71	Mozavaptan
72	Cyproterone Acetate

73	Sodium 4-Aminosalicylate
74	GSK1292263
75	SGC 0946
76	LY2157299
77	IPA-3
78	Esomeprazole Sodium
79	DMH1
80	Ozagrel
81	AZD4547
82	GW9508
83	VE-821
84	NSC 405020
85	GNF-2
86	TPCA-1
87	T0901317
88	PD0325901
89	PYR-41
90	U0126-EtOH
91	LDN-212854
92	Ki16425
93	C646
94	Oxcarbazepine
95	Mdivi-1
96	MRS 2578
97	AGI-5198
98	LY2603618
99	BTB06584
100	NPS-2143
101	Veliparib (ABT-888)
102	Dalcetrapib (JTT-705, RO4607381)
103	Vandetanib (ZD6474)
104	Istradefylline
105	Iniparib (BSI-201)
106	Dabrafenib (GSK2118436)
107	Finasteride
108	Tyrphostin AG 879
109	Cilomilast
110	TAE226 (NVP-TAE226)
111	Ozagrel HCl

112	Vildagliptin (LAF-237)
113	Dynasore
114	Piceatannol
115	Quizartinib (AC220)
116	Tenovin-6
117	Enzastaurin (LY317615)
118	CGP 57380
119	Bisoprolol fumarate
120	Bosutinib (SKI-606)
121	S3I-201
122	HA14-1
123	TG100-115
124	ADL5859 HCl
125	Voriconazole
126	BIBR 1532
127	Thiazovivin
128	Anastrozole
129	SB743921
130	EUK 134
131	Bergenin
132	SN-38
133	CP-91149
134	Wnt-C59 (C59)
135	NU7026
136	BAM7
137	ZM 306416
138	(+)-JQ1
139	GW9662
140	KPT-185
141	Pifithrin-μ
142	Batimastat (BB-94)
143	Sertraline HCl
144	OG-L002
145	MK-1775
146	Costunolide
147	AT101
148	GSK690693
149	Tropicamide
150	BMS-707035

151	Raltegravir (MK-0518)
152	EX 527 (Selisistat)
153	CCT128930
154	Pomalidomide
155	AS-252424
156	Tie2 kinase inhibitor
157	Ouabain
158	Ranitidine
159	SKI II
160	Fluvastatin Sodium
161	Propranolol HCl
162	Erastin
163	Ifenprodil Tartrate
164	KPT-276
165	AZD2461
166	KPT-330
167	AGI-6780
168	SGI-1027
169	Atglistatin
170	Suvorexant (MK-4305)
171	SRT1720
172	4EGI-1
173	Exemestane
174	NSC 23766
175	2-Methoxyestradiol (2-MeOE2)
176	Palbociclib (PD-0332991) HCl
177	EHop-016
178	PF-573228
179	ABT-199 (GDC-0199)
180	Memantine HCl
181	PTC-209
182	Trimebutine
183	CK-636
184	SGI-1776 free base
185	AZD7545
186	URB597
187	GW0742
188	TAK-875
189	Pacritinib (SB1518)

190	KX2-391
191	PluriSIn #1 (NSC 14613)
192	Crenolanib (CP-868596)
193	Enzalutamide (MDV3100)
194	PFI-1 (PF-6405761)
195	Dapagliflozin
196	Maraviroc
197	Nebivolol
198	I-BET151 (GSK1210151A)
199	VX-809 (Lumacaftor)
200	Apoptosis Activator 2
201	Naproxen
202	Bosentan Hydrate
203	Acadesine
204	E-64
205	Captopril
206	Selumetinib (AZD6244)
207	Tolvaptan
208	PD184352 (CI-1040)
209	OSI-906 (Linsitinib)
210	Canagliflozin
211	CP-673451
212	Sirtinol
213	Abitrexate (Methotrexate)
214	SAR131675
215	Pralatrexate
216	BML-190
217	TWS119
218	IKK-16 (IKK Inhibitor VII)
219	Enalaprilat Dihydrate
220	Triamterene
221	Clemastine Fumarate
222	Fingolimod (FTY720) HCl
223	Amlodipine
224	PP2
225	CHIR-124
226	Temsirolimus (CCI-779, NSC 683864)
227	YO-01027
228	Trichostatin A (TSA)

229	PAC-1
230	PHA-665752
231	VE-822
232	SB203580
233	EPZ-6438
234	KU-55933 (ATM Kinase Inhibitor)
235	CGK 733
236	WZ4002
237	WZ4003
238	TAK-700 (Orteronel)
239	Loxistatin Acid (E-64C)
240	Zibotentan (ZD4054)
241	Pyrimethamine
242	RKI-1447
243	UNC2250
244	SMI-4a
245	SB415286
246	PRT062607 (P505-15, BIIB057) HCl
247	Torcetrapib
248	MK-2206 2HCl
249	ML130 (Nodinitib-1)
250	PF-04217903
251	GW441756
252	Varespladib (LY315920)
253	ML161
254	MK-2866 (GTx-024)
255	Ticagrelor
256	Letrozole
257	GW2580
258	Zosuquidar (LY335979) 3HCl
259	KU-60019
260	LY2228820
261	MLN2238
262	Org 27569
263	Oxymetazoline HCl
264	DMXAA (Vadimezan)
265	Anacetrapib (MK-0859)
266	AM1241
267	Embelin

268	Toremifene Citrate
269	GSK2656157
270	Felodipine
271	(+)-Bicuculline
272	Ticlopidine HCl
273	SANT-1
274	Ispinesib (SB-715992)
275	BTZ043 Racemate
276	AZD7762
277	AVL-292
278	Pimobendan
279	DBeQ
280	Formoterol Hemifumarate
281	CNX-774
282	Lovastatin
283	4μ8C
284	Lafutidine
285	AZ191
286	(-)-Parthenolide
287	JSH-23
288	Pramipexole
289	RepSox
290	Bazedoxifene HCl
291	Golgicide A
292	LDE225 (NVP-LDE225,Erismodegib)
293	Ridaforolimus (Deforolimus, MK-8669)
294	LY2784544
295	SNS-314 Mesylate
296	BGJ398 (NVP-BGJ398)
297	Irinotecan
298	OSI-420
299	Dutasteride
300	Apigenin
301	Rigosertib (ON-01910)
302	Forskolin
303	Rolipram
304	Bupivacaine HCl
305	UNC669
306	Tioxolone

307	PF-4708671
308	5-hydroxymethyl Tolterodine (PNU 200577, 5-HMT, 5-HM)
309	XAV-939
310	SB742457
311	Cinacalcet HCl
312	Linagliptin
313	Etomidate
314	Entacapone
315	AG-14361
316	Moclobemide (Ro 111163)
317	LY411575
318	GDC-0152
319	OC000459
320	NLG919
321	Levosulpiride
322	Imatinib (STI571)
323	DCC-2036 (Rebastinib)
324	XL335
325	Nilvadipine
326	CHIR-98014
327	GW4064
328	PF-5274857
329	GDC-0068
330	JNJ-1661010
331	VU 0364770
332	U-104
333	Daunorubicin HCl
334	PF-562271
335	AZD3463
336	IOX2
337	IMD 0354
338	CRT0044876
339	TCID
340	LB42708
341	Necrostatin-1
342	Empagliflozin (BI 10773)
343	SU11274
344	Bortezomib (PS-341)
345	YM155 (Sepantronium Bromide)

346	Lenalidomide (CC-5013)
347	Ivacaftor (VX-770)
348	AUY922 (NVP-AUY922)
349	Agomelatine
350	17-AAG (Tanespimycin)
351	SP600125
352	CEP-18770 (Delanzomib)
353	Aprepitant
354	Fluvoxamine maleate
355	Oligomycin A
356	Ginkgolide A
357	Cryptotanshinone
358	ICG-001
359	Stattic
360	SC144
361	SRPIN340
362	Trelagliptin
363	Panobinostat (LBH589)
364	VX-680 (Tozasertib, MK-0457)
365	GDC-0941
366	OSU-03012 (AR-12)
367	GSK690693
368	Everolimus (RAD001)
369	MK-8245
370	Aniracetam
371	Doxazosin Mesylate
372	Ginkgolide B
373	Tosedostat (CHR2797)
374	Rebamipide
375	Rasagiline Mesylate
376	PD128907 HCl
377	Apatinib
378	ADX-47273
379	AZ 3146
380	VU 0357121
381	(-)-MK 801 Maleate
382	Mirabegron
383	AP26113
384	Birinapant

385	AZD1981
386	LDK378
387	(S)-crizotinib
388	ZM 447439
389	BX-912
390	Tadalafil
391	Elvitegravir (GS-9137, JTK-303)
392	Fostamatinib (R788)
393	GSK J4 HCl
394	TCS 359
395	Carvedilol
396	Naftopidil
397	ML133 HCl
398	T0070907
399	Gliquidone
400	SC-514
401	ZCL278
402	Caffeic Acid Phenethyl Ester
403	VU 0364439
404	SB431542
405	Odanacatib (MK-0822)
406	Celecoxib
407	Etodolac
408	Isotretinoin
409	Stavudine (d4T)
410	VX-745
411	GSK429286A
412	SB408124
413	H 89 2HCl
414	Mubritinib (TAK 165)
415	BMS-378806
416	Ki16198
417	AZ20
418	AMG-517
419	NMS-873
420	Sorafenib
421	NH125
422	Sal003
423	Tariquidar

424	Lomeguatrib
425	BI 2536
426	Imidapril HCl
427	GSK461364
428	Gliclazide
429	Sotrastaurin
430	BI-D1870
431	Go 6983
432	MNS (3,4-Methylenedioxy-β-nitrostyrene, MDBN)
433	THZ1

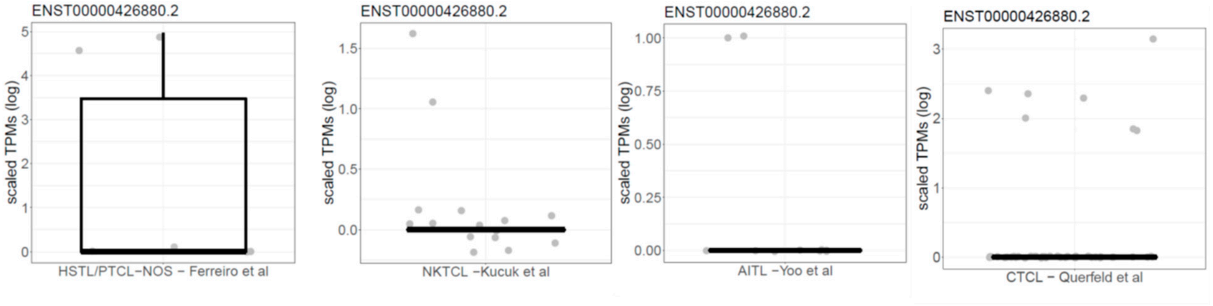

Figure S1. *H4R* mRNA expression (ENST00000426880.2 transcript variant 2, short isoform) in tumors of TCL patients. The transcriptional datasets of tumors from patients with AITL (accession number: SRP029591, n=10) [28], hepatosplenic TCL (HSTL)/PTCL-NOS (accession number: SRP039591, n=6) [30], NKTCL (accession number: SRP049695, n=15) [31], and CTCL (accession number: SRP139926, n=47) [32] were obtained from the NCBI Sequence Read Archive and analyzed as indicated in materials and methods. TPMs: transcripts per million.

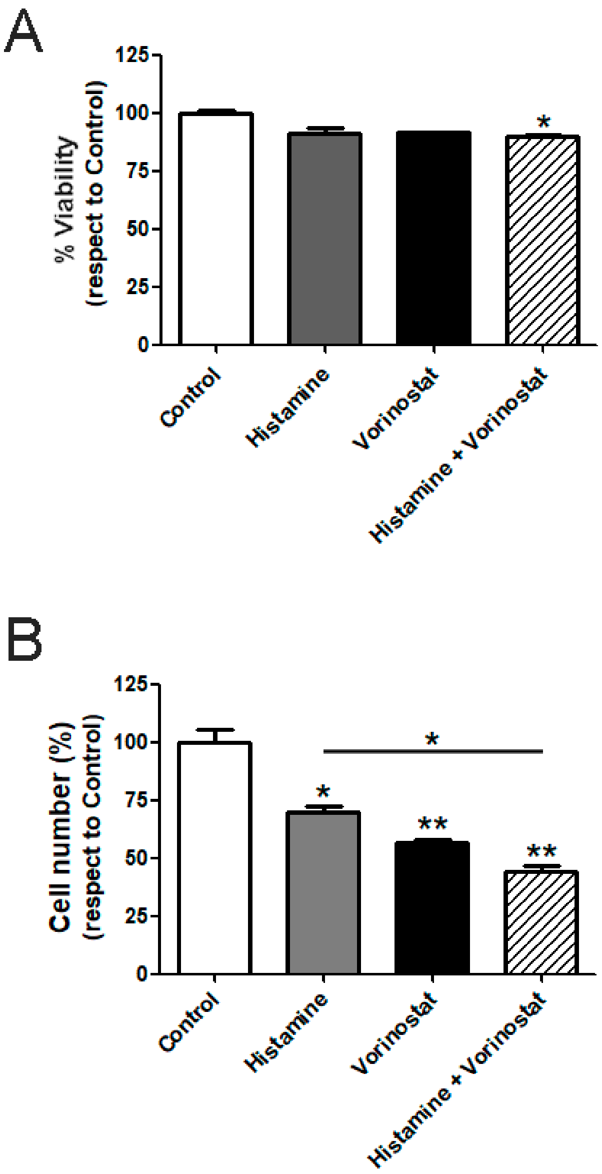

Figure S2: Effect of the combination of histamine and vorinostat on HuT78 cell viability and cell number. HuT78 cells were pre-incubated for 24 h in serum-free RPMI medium and then were left untreated (control) or were treated with histamine (10 μM) and/or Vorinostat (1 μM) for 48 h in complete medium, as indicated. A) Cell viability was evaluated by Cell Titer Blue Assay. B) The cell number was counted using a Neubauer chamber. Measurements were performed in triplicates for each condition and data are expressed as mean ± SEM (n=3 independent experiments). * indicates $p<0.05$, ** indicates $p<0.01$ compared with Control.
